# Assessment of Iodine Status in Pregnant Women: Diagnostic Performance of Spot Urinary Iodine Indices Compared with 24-h Urinary Iodine Excretion

**DOI:** 10.3390/nu18050835

**Published:** 2026-03-04

**Authors:** Emre Altuğ, Gamze Dur, Nazli Sensoy, Aysen Mert, Halit Bugra Koca

**Affiliations:** 1 Güzelsu Family Health Center, Gerger 02702, Türkiye; dr.emrealtug@gmail.com; 2 Department of Family Medicine, Faculty of Medicine, Afyonkarahisar Health Sciences University, Afyonkarahisar 03030, Türkiye; nazli.sensoy@afsu.edu.tr (N.S.); aysen.mert@afsu.edu.tr (A.M.); 3 Department of Medical Biochemistry, Faculty of Medicine, Afyonkarahisar Health Sciences University, Afyonkarahisar 03030, Türkiye; bugrakoca@yahoo.com

**Keywords:** pregnancy, iodine status, iodine deficiency, 24 h urinary iodine excretion (24h-UIE), urinary iodine concentration (UIC), iodine-to-creatinine ratio (UIC/UCr), maternal nutrition, thyroid function in pregnancy, universal salt iodization, nutritional biomarker

## Abstract

**Background**: Adequate iodine intake during pregnancy is essential for optimal maternal thyroid function and fetal neurodevelopment. Although universal salt iodization has been implemented in Turkey, pregnant women may remain vulnerable to iodine insufficiency. This study aimed to evaluate maternal thyroid function in relation to iodine status, and to comprehensively compare the diagnostic performance of spot urinary indices and creatinine-adjusted measures against measured 24 h urinary iodine excretion (24h-UIE) in pregnant women. **Methods**: A total of 227 pregnant women attending family health centers in Afyonkarahisar, Turkey, provided both spot urine samples and complete 24 h urine collections. Urinary iodine concentration (UIC), creatinine-corrected UIC (UIC/UCr), and 24h-UIE were measured. Thyroid function tests were interpreted using trimester-specific reference ranges. Correlations between urinary indices were assessed, and ROC analyses were performed using 24h-UIE as the operational reference. A structured questionnaire evaluated iodine-related dietary knowledge and salt-use practices. **Results**: The median spot UIC was 59.0 µg/L, indicating insufficient recent iodine intake at the population level. Based on 24h-UIE, 70% of participants had excretion levels below the Estimated Average Requirement (EAR) threshold (<144 µg/day). Spot UIC showed a weak correlation with 24h-UIE (rho = 0.270, *p* < 0.001), whereas UIC/UCr demonstrated a stronger correlation (rho = 0.491, *p* < 0.001). In ROC analyses, UIC/UCr yielded a significantly higher AUC than spot UIC (0.774 [95% CI: 0.707–0.841] vs. 0.670 [95% CI: 0.593–0.748]; DeLong *p* = 0.016). Overt hypothyroidism was not observed; subclinical hypothyroidism was present in 16.3% of participants. While no overall association was found between iodine indices and thyroid status, in the first trimester, those with subclinical hypothyroidism had higher 24h-UIE medians than euthyroid peers (134.2 vs. 100.3 µg/day, *p* = 0.037), although both groups remained below the EAR threshold. Knowledge regarding iodine-rich foods and iodized salt use was limited among the study population. **Conclusions**: Iodine insufficiency remains highly prevalent among pregnant women in this region despite salt iodization. While spot UIC alone showed limited agreement with 24h-UIE, creatinine-adjusted UIC may offer improved interpretability under conditions of variable urine dilution. Preserved thyroid function in the presence of iodine insufficiency highlights the silent nature of this condition during pregnancy. Strengthened pregnancy-specific iodine surveillance and targeted antenatal education are warranted.

## 1. Introduction

Substantial global progress has been made in the control of iodine deficiency disorders thanks to the widespread implementation of salt iodization programs. However, despite these major public health achievements, ensuring adequate iodine intake remains a critical challenge for vulnerable populations, particularly pregnant women, due to their significantly increased physiological requirements [1,2]. Indeed, global surveillance and regional assessments continue to report mild-to-moderate iodine insufficiency among pregnant women, even in regions with established control programs such as parts of Europe [2,3,4]. Consequently, UNICEF continues to identify iodine deficiency as a leading cause of preventable cognitive impairment, emphasizing the heightened vulnerability during pregnancy and early childhood [5].

Iodine is an essential substrate for thyroid hormone synthesis, and adequate maternal thyroxine (T4) availability is critical for fetal brain development, particularly in early gestation when the fetus is largely dependent on maternal thyroid hormone supply [6,7]. Pregnancy increases iodine requirements due to a ~50% increase in maternal thyroid hormone production to maintain euthyroidism [6,7], alongside physiological adaptations such as increased renal perfusion and glomerular filtration, which can increase renal iodine losses [6,8]. Consequently, even mild iodine deficiency during pregnancy may be associated with suboptimal maternal thyroid function and adverse offspring neurodevelopmental outcomes, with potentially important implications at the population level [1,2,9]. At the individual level, measured 24 h urinary iodine excretion (24h-UIE) from a complete 24 h urine collection is widely regarded as the reference method for assessing recent iodine intake; however, 24 h collections are logistically burdensome and susceptible to incomplete collection and timing-related errors in both routine practice and epidemiologic studies [9,10,11]. Accordingly, the World Health Organization (WHO) recommends median urinary iodine concentration (UIC) from spot urine samples for population-level monitoring, with pregnancy-specific cut-offs (median UIC 150–249 µg/L indicating adequate iodine intake) [1]. Interpretation of spot UIC in pregnancy is complicated by pregnancy-related changes in renal physiology and variation in urine dilution. To mitigate dilution-related variability, creatinine-adjusted indices—most commonly the urinary iodine-to-creatinine ratio (UIC/UCr)—have been proposed to account for urine dilution when interpreting spot iodine measures [11,12,13]. In particular, some studies report that the urinary iodine-to-creatinine ratio (UIC/UCr) may better reflect 24 h urinary iodine excretion (24h-UIE) than spot UIC alone in pregnant women, although the optimal metric for assessment remains debated [10,11,12,13]. Furthermore, assessing dietary sources of iodine and maternal knowledge and practices regarding iodized salt is important for elucidating determinants of iodine insufficiency beyond biochemical monitoring alone [9,14,15,16]. Turkey has historically experienced moderate-to-severe iodine deficiency [17,18]. Although the iodization of table salt has been mandatory in Turkey since 1998 [19], evidence suggests that pregnant women remain vulnerable to iodine insufficiency across multiple regions [15,17,18,20,21]. However, studies directly comparing spot urinary iodine indices with measured 24 h urinary iodine excretion in pregnant women, particularly within this setting, remain relatively limited [10,12,13]. Given that maternal thyroid function is the fundamental biological basis for identifying the clinical impact of iodine deficiency, the primary objective of this study was to evaluate the diagnostic performance of 24h-UIE, median spot UIC, and UIC/UCr strictly within the context of thyroid hormone status. Specifically, we aimed to investigate how these various urinary indices reflect maternal thyroid function across different trimesters, and ultimately, to determine their precision in identifying adequate recent iodine intake among pregnant women. Secondarily, the study aimed to assess iodine-related dietary habits and knowledge regarding iodized salt to provide a more comprehensive evaluation of iodine status in pregnancy.

## 2. Materials and Methods

### 2.1. Study Design and Setting

This analytical cross-sectional study was conducted between 1 March and 1 September 2022 in six Family Health Centers (FHCs) located in the urban area of Afyonkarahisar, Turkey. Centers were selected using a purposive sampling method to represent different socioeconomic strata within the study area. Reporting of this cross-sectional study adheres to the STROBE recommendations for observational research [22].

### 2.2. Participants and Eligibility Criteria

The target population consisted of pregnant women aged 18–49 years attending routine antenatal care at the participating FHCs. Gestational age (1–40 weeks) was ascertained based on the first day of the last menstrual period and confirmed by first-trimester ultrasonography records.

Exclusion criteria were: (i) documented or self-reported history of thyroid disease; (ii) current use of thyroid medication; (iii) use of iodine-containing vitamin/mineral supplements; (iv) multiple pregnancy; (v) chronic systemic disease (e.g., chronic kidney disease, diabetes mellitus, autoimmune disorders); and (vi) conception via assisted reproductive techniques. These criteria were applied to obtain a relatively homogeneous population for comparing urinary iodine indices.

### 2.3. Sample Size and Study Flow

The sample size was estimated a priori using G*Power software (version 3.1; Heinrich-Heine-Universität Düsseldorf, Düsseldorf, Germany) [23]. Because trimester-stratified comparisons were planned, the calculation was based on a one-way omnibus comparison across three groups (trimester strata) (F tests, one-way ANOVA fixed effects, omnibus), assuming a medium effect size (Cohen’s f = 0.25), a two-sided Type I error rate (α) of 0.05, and 90% power, yielding a minimum required total sample size of 207 participants. To accommodate non-evaluable participants (e.g., incomplete 24 h urine collections or missing key variables), the target sample size was inflated by approximately 15%, and 240 pregnant women were initially recruited. After prespecified data-quality checks and eligibility confirmation, 13 participants were excluded due to missing key data and/or incomplete 24 h urine collections, resulting in a final analytical sample of 227 participants.

### 2.4. Data Collection and Questionnaire

Data were collected during scheduled antenatal visits by a single trained physician-researcher using a structured, interviewer-administered questionnaire developed from the relevant literature on iodine nutrition in pregnancy.

The questionnaire included: (i) sociodemographic characteristics (age, education, employment status, household income, and place of residence); (ii) obstetric and medical history (gravidity, parity, gestational week, prior pregnancy outcomes, and smoking status); (iii) salt-related behaviors (use of iodized vs. non-iodized salt, timing of salt addition during cooking, and storage conditions); and (iv) dietary patterns related to iodine, including the frequency of consumption of commonly cited goitrogenic foods (e.g., turnip, radish, cabbage, kale/black cabbage, broccoli, cauliflower, millet) [24] and dietary iodine sources (e.g., dairy products, seafood, meat products, legumes, green vegetables) [1,21], as well as self-reported knowledge regarding the iodine content of these foods.

### 2.5. Biological Sample Collection

All biological samples were collected following a standardized protocol to minimize pre-analytical variability. Blood Sampling: Venous blood samples (3–5 mL) were obtained from each participant in the morning after an overnight fast (minimum 8 h) to assess thyroid function and renal parameters under basal conditions. Urine Sampling (Dual-Collection Protocol): To compare iodine assessment methods, participants followed a two-step urine collection procedure:

1. Spot Urine: A fasting mid-stream urine sample was collected during the morning clinic visit, concurrently with blood sampling. This sample served as the source for the ‘Spot UIC’ measurement to ensure temporal alignment with thyroid function tests.
2. Twenty-four-hour Urine: Participants were instructed to discard the first morning void on the start day (which corresponded to the spot sample timepoint), collect all subsequent urine for the next 24 h (including the first void of the following morning). Samples were kept in a cool, dark environment during collection. Upon return, the total 24 h volume was recorded, samples were homogenized, aliquoted and stored at −80 °C until analysis.

### 2.6. Laboratory Measurements

Serum thyroid-stimulating hormone (TSH) and free thyroxine (fT4) levels were measured using an electrochemiluminescence immunoassay (ECLIA) on a Cobas 8000 analyzer (Roche Diagnostics, Mannheim, Germany). Serum creatinine was measured using a calibrated colorimetric (Jaffe) method on the same platform. Urine Analysis: Urinary iodine concentration (UIC, µg/L) in both spot and 24 h urine aliquots was measured using inductively coupled plasma–mass spectrometry (ICP–MS) on a NEXION 2000 instrument (PerkinElmer, Waltham, MA, USA). Urinary creatinine concentration (UCr, mg/dL) was determined using a colorimetric method on an Abbott Architect c8000 analyzer (Abbott Diagnostics, Abbott Park, IL, USA).

### 2.7. Thyroid Function Definitions

Thyroid function was classified using trimester-specific reference ranges based on local laboratory standards and national/international guidance [21,25]: TSH (0.1–2.5 mU/L for the 1st trimester; 0.2–3.0 mU/L for the 2nd trimester; and 0.3–3.0 mU/L for the 3rd trimester) and fT4 (0.8–1.53 ng/dL for the 1st trimester; 0.7–1.20 ng/dL for the 2nd and 3rd trimesters).

### 2.8. Urinary Iodine Indices and Definitions

For each participant, three urinary iodine indices were derived:

- Spot UIC (µg/L): Measured directly in the spot urine sample.
- Urinary Iodine-to-Creatinine ratio (UIC/UCr, µg/g): Calculated by dividing spot UIC (µg/L) by spot UCr expressed in g/L (converted from mg/dL) to adjust for urine dilution.
- Twenty-four-hour Urinary Iodine Excretion (24h-UIE, µg/day): Calculated as UIC in the 24 h sample (µg/L) multiplied by total 24 h urine volume (L/day).

Population Iodine Status by Spot UIC: Consistent with WHO guidelines, the median spot UIC was used exclusively as the primary indicator for assessing population-level iodine status, with adequacy defined as a population median of 150–249 µg/L [1]. In accordance with epidemiological principles, WHO thresholds (such as the reference low value of <50 µg/L, 50–99 µg/L, etc.) were strictly interpreted as criteria for evaluating the overall population median. These values were not utilized as cut-offs for categorizing individual participant results or for calculating frequency distributions.

Iodine status based on 24h-UIE: In accordance with epidemiological principles for assessing population-level prevalence of inadequate nutrient intake, the Estimated Average Requirement (EAR) for pregnant women (160 µg/day based on the Institute of Medicine of the USA) [26] was utilized. Assuming approximately 90% of ingested iodine is excreted in urine, a 24h-UIE value of <144 µg/day was used as the operational threshold to estimate the proportion of the population at risk of iodine inadequacy.

UIC/UCr ratio: As there is no universally accepted pregnancy-specific cut-off for UIC/UCr, this index was analyzed primarily as a continuous variable and compared with 24h-UIE using correlation and ROC analyses.

### 2.9. Statistical Analysis

All statistical analyses were performed using IBM SPSS Statistics for Windows, version 26.0 (IBM Corp., Armonk, NY, USA). Normality was assessed via the Shapiro–Wilk test and visual inspection of Q-Q plots. Non-normally distributed continuous variables (including UIC, UIC/UCr, 24h-UIE, TSH, fT4, UCr, and eGFR) are presented as median (interquartile range, IQR); while normally distributed data (e.g., BMI) are reported as mean ± standard deviation (SD). Categorical variables are summarized as frequencies and percentages. The Mann–Whitney U test was used for two-group comparisons. Trimester-based differences were examined using the Kruskal–Wallis test; when the overall *p*-value was <0.05, pairwise post hoc comparisons were performed using Dunn’s test with Bonferroni adjustment. Associations between iodine-related parameters (UIC, UIC/UCr, 24h-UIE, UCr, eGFR, and BMI) were evaluated using Spearman’s rank correlation coefficient in the full sample and separately by trimester. Receiver operating characteristic (ROC) curve analysis was used to assess the ability of spot UIC and UIC/UCr to discriminate between women with inadequate and adequate recent iodine intake. Inadequacy was defined using 24h-UIE < 144 µg/day as the reference standard, a threshold reflecting the 90% urinary excretion of the 160 µg/day (EAR) for pregnancy. The areas under the curve (AUCs) with 95% confidence intervals (CIs) and corresponding *p*-values were reported. AUCs were compared for two related ROC curves using the DeLong test [27] in MedCalc^®^ version 23.3.7. All tests were two-sided and a *p*-value < 0.05 was considered statistically significant.

### 2.10. Ethical Considerations

The study was conducted in accordance with the Declaration of Helsinki and was approved by the Non-Interventional Clinical Research Ethics Committee of Afyonkarahisar Health Sciences University (Date: 4 February 2022; Decision No: 2022/76). Institutional permission was obtained from the Afyonkarahisar Provincial Health Directorate (Date: 25 February 2022; Decision No: 2022/8). All participants were informed about the purpose and procedures of the study, and written informed consent was obtained from each subject prior to enrollment. This study was supported by the Scientific Research Projects Unit of Afyonkarahisar Health Sciences University (Project Code: 22.TUS.001).

## 3. Results

### 3.1. Participant Characteristics

The final analytical sample comprised 227 pregnant women, including 63 (27.8%) in the first trimester, 78 (34.4%) in the second trimester, and 86 (37.8%) in the third trimester. The median age of participants was 27.0 years (range: 18–41 years), and the mean body mass index (BMI) was 26.95 ± 4.57 kg/m^2^. Regarding sociodemographic characteristics, 41.0% (*n* = 93) of the women were university graduates, and 75.8% (*n* = 172) were not engaged in paid employment. The majority resided in the city center (92.5%, *n* = 210). Household income was categorized as low (minimum wage or below), medium (above the minimum wage), or high (more than twice the minimum wage); most participants belonged to the medium-income group (90.3%, *n* = 205). At the time of assessment, 5.7% (*n* = 13) of participants were active smokers, while 7.9% (*n* = 18) reported smoking cessation after becoming pregnant.

### 3.2. Consumption Frequency and Iodine-Related Knowledge

When asked about iodine deficiency and its health consequences, 76.7% (*n* = 174) of participants stated they had never received any information on the topic. Regarding household salt use, 59.9% (*n* = 136) reported using iodized salt, 11.9% (*n* = 27) used non-iodized salt, 27.3% (*n* = 62) were unaware of the salt type, and two women (0.9%) reported not using any salt. Given that table salt in Turkey is mandatorily iodized (legally required to contain 25–40 mg of potassium iodate per kilogram), participants unaware of their salt type were grouped with iodized salt users. However, non-iodized alternatives (e.g., rock salt, gourmet salts) remain commercially accessible; consequently, 11.9% of participants reported specifically consuming non-iodized salt.

Consequently, 87.2% (*n* = 198) were classified as ‘iodized salt users’ and 12.8% (*n* = 29) as ‘non-iodized salt users’ (including those restricting salt). Iodized salt usage was not significantly associated with educational level (*p* = 0.415) or household income (*p* = 0.449).

Table 1 summarizes the consumption frequency of selected goitrogenic and iodine-rich foods and maternal knowledge regarding their iodine content. Dairy products—potentially the major dietary source of iodine—and green vegetables were the most frequently consumed items, with 96.5% (219/227) of participants reporting at least weekly consumption for both. Meat products (84.1%, 191/227) and legumes (80.6%, 183/227) were also commonly consumed, whereas weekly seafood consumption was reported by 47.1% (107/227). Across all listed items, most participants reported that they did not know whether the foods were sources of iodine (range: 74.4–91.2%; Table 1).

### 3.3. Thyroid/Renal Parameters and Urinary Iodine Indices Stratified by Trimester

Thyroid and renal parameters, along with urinary iodine indices stratified by trimester, are presented in Table 2. Statistically significant trimester-specific differences were observed for both thyroid and renal function markers. TSH levels were significantly higher (*p* = 0.012) and fT4 levels were lower (*p* < 0.001) in the advanced gestational groups compared to the first trimester group. Regarding renal function, spot UCr levels differed significantly across groups (*p* = 0.008), and eGFR was significantly higher in the second and third trimesters compared with the first trimester (*p* < 0.001).

Based on trimester-specific reference ranges, 37/227 (16.3%) participants met the criteria for subclinical hypothyroidism (elevated TSH with normal fT4), with prevalences of 22.2% (14/63), 9.0% (7/78), and 18.6% (16/86) in the first, second, and third trimesters, respectively; however, these prevalence rates did not differ significantly across trimesters (*p* = 0.081). No participant met the criteria for overt hypothyroidism; overt hyperthyroidism was observed in 2/227 (0.9%) and subclinical hyperthyroidism in 1/227 (0.4%). Overall, 166/227 (73.1%) participants were euthyroid.

Regarding urinary iodine indices, the overall median spot UIC was 59.0 (IQR 54.6) µg/L. Although median spot UIC values differed numerically among the trimester groups (1st: 67.5 µg/L; 2nd: 71.6 µg/L; 3rd: 53.2 µg/L), these differences were not statistically significant (*p* = 0.121). Similarly, neither the creatinine-corrected index (UIC/UCr; *p* = 0.314) nor the 24h-UIE (*p* = 0.302) showed significant variation between the trimester groups.

To comprehensively evaluate the relationship between iodine status and thyroid function, urinary iodine indices were categorized by thyroid status (Euthyroid vs. Subclinical Hypothyroidism), as presented in Table 3. Overall, there were no significant differences in median spot UIC, UIC/UCr, or 24h-UIE between the euthyroid and subclinical hypothyroid groups. However, trimester-specific sub-analysis revealed that during the first trimester, pregnant women with subclinical hypothyroidism exhibited a significantly higher median 24h-UIE compared to their euthyroid peers (*p* = 0.037), although both groups remained below the EAR adequacy threshold.

### 3.4. Population Iodine Status and 24h-UIE Adequacy

To evaluate the prevalence of iodine inadequacy, we applied the EAR-adjusted 24h-UIE threshold of <144 µg/day, which accounts for the standard 90% urinary excretion of the 160 µg/day intake requirement during pregnancy [26]. Based on this reference standard, 70.0% (159/227) of the participants exhibited excretion levels below the adequacy threshold. Consequently, only 30.0% (68/227) of the pregnant women demonstrated adequate recent iodine intake. When stratified by trimester, the proportion of women with adequate iodine levels was 27.0% (17/63) in the first trimester, 33.3% (26/78) in the second trimester, and 29.1% (25/86) in the third trimester. The adequacy rates did not differ significantly across the three trimesters (*p* = 0.697).

At the population level, the median spot UIC was 59.0 µg/L, which is markedly below the WHO adequacy threshold of 150 µg/L for pregnant populations, indicating insufficient recent iodine intake.

### 3.5. Correlation Analyses with 24h-UIE as the Reference Standard for Population Iodine Intake

Spearman correlations are presented in Table 4. In the overall sample, 24h-UIE correlated with spot UIC (rho = 0.270, *p* < 0.001) and with UIC/UCr (rho = 0.491, *p* < 0.001). In trimester-stratified analyses, the correlation between spot UIC and 24h-UIE was not statistically significant in the second trimester (rho = 0.125, *p* = 0.275), whereas UIC/UCr remained correlated with 24h-UIE across all trimesters (1st: rho = 0.394, *p* = 0.001; 2nd: rho = 0.553, *p* < 0.001; 3rd: rho = 0.533, *p* < 0.001). Spot UIC was correlated with spot UCr (overall rho = 0.489, *p* < 0.001), and UIC/UCr was inversely correlated with UCr (overall rho = −0.521, *p* < 0.001) (Table 4).

### 3.6. ROC Curve Analyses

**Figure 1:**
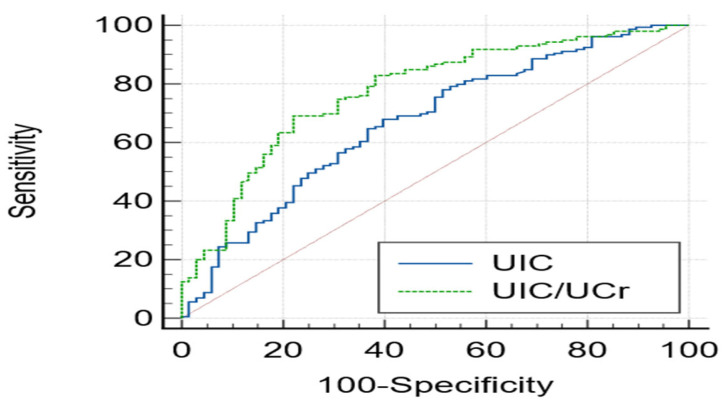
Receiver operating characteristic (ROC) curves of spot urinary iodine concentration (UIC) and the urinary iodine-to-creatinine ratio (UIC/UCr) for identifying pregnant women with adequate recent iodine intake, defined as 24 h urinary iodine excretion (24h-UIE) ≥ 144 µg/day. The area under the curve (AUC) for spot UIC was 0.670 (95% CI: 0.593–0.748; *p* < 0.001), whereas the AUC for UIC/UCr was 0.774 (95% CI: 0.707–0.841; *p* < 0.001). The diagnostic performance of UIC/UCr was significantly superior to spot UIC alone (DeLong test, *p* = 0.016).

ROC analysis was performed to evaluate the ability of spot UIC and UIC/UCr to discriminate pregnant women with adequate recent iodine intake, defined as 24h-UIE ≥ 144 µg/day (Figure 1). The area under the curve (AUC) was 0.670 (95% CI: 0.593–0.748; *p* < 0.001) for spot UIC and 0.774 (95% CI: 0.707–0.841; *p* < 0.001) for UIC/UCr. The pairwise comparison of the two correlated ROC curves demonstrated that the diagnostic performance of UIC/UCr was significantly superior to that of spot UIC alone (DeLong test, *p* = 0.016) [27].

## 4. Discussion

The present study provides a comprehensive evaluation of iodine status among pregnant women in Afyonkarahisar, Turkey, using a dual-collection protocol that allowed direct comparison of spot urinary indices with measured 24h-UIE. The median spot UIC of the study population was 59.0 µg/L, which is well below the WHO adequacy threshold of 150 µg/L for pregnant populations. Furthermore, when rigorously evaluated using the reference standard 24h-UIE, exactly 70.0% of the participants exhibited excretion levels strictly below the EAR-derived adequacy threshold (<144 µg/day). The overall median 24h-UIE observed in our cohort (111.2 µg/day) fell far short of this critical physiological benchmark. Taken together, these findings confirm that the population faces a genuine, widespread nutritional deficit, consistently failing to meet the foundational daily iodine requirements for healthy pregnancy. Beyond these biochemical indicators of widespread population-level inadequacy, our questionnaire data revealed marked gaps in maternal knowledge regarding dietary iodine sources. This suggests that behavioral and informational factors fundamentally contribute to the persistence of iodine insufficiency, overshadowing the potential benefits of long-standing salt iodization policies.

The overall median UIC of 59.0 µg/L observed in our study population is markedly below the WHO-defined adequacy threshold of 150 µg/L for pregnancy, indicating widespread iodine insufficiency in this region. Although Turkey has implemented mandatory table salt iodization since 1998 [18,19]—requiring all table salt to be fortified with 25–40 mg of potassium iodate per kilogram—our findings highlight a persistent nutritional vulnerability. Indeed, while 87.2% of our cohort were classified as iodized salt users—closely mirroring national surveys where over 85% of households claim to use fortified salt—a comprehensive nationwide titration study [18] previously demonstrated that only 56.5% of household salt samples actually comply with adequate iodine levels (>15 ppm) due to production inconsistencies and improper storage. Despite this extensive population-wide policy, our results are highly consistent with accumulating national and regional evidence demonstrating that pregnant women remain a uniquely vulnerable subgroup, frequently failing to meet their abruptly increased physiological iodine requirements through fortified salt alone [17,18,20,21]. Notably, the median UIC in our cohort is lower than the value reported in recent nationwide data by Vural et al. [20], suggesting pronounced regional heterogeneity and the persistence of iodine-inadequate pockets. Importantly, this pattern aligns with observations from other settings with long-standing universal salt iodization programs, where iodine sufficiency in school-aged children does not necessarily translate into adequacy during pregnancy [28,29]. Kuay et al. demonstrated that, despite adequate iodine status among school-aged children, pregnant women exhibited iodine inadequacy, underscoring the critical limitation of extrapolating population-level sufficiency indicators to physiologically high-risk groups such as pregnant women [28]. Together, these findings emphasize that pregnancy-specific iodine surveillance is essential, even in countries considered iodine sufficient at the general population level.

Beyond policy implementation gaps, behavioral and dietary factors further compound this discrepancy. Recent data from Turkey indicate that salt-restriction practices during pregnancy—often encouraged for perceived cardiovascular or obstetric benefits—may inadvertently reduce iodine intake in the absence of alternative iodine sources or supplementation [15]. This is particularly critical given that dairy products often serve as a primary dietary iodine source, effectively complementing or even exceeding the contribution of iodized salt in many regions. Consistent with this, our survey findings revealed limited maternal awareness of dietary iodine sources and iodized salt usage, suggesting that inadequate knowledge exacerbates physiological vulnerability and fundamentally undermines the effectiveness of national iodization policies.

Despite the high prevalence of low urinary iodine, overt hypothyroidism was not observed. While median thyroid hormone levels remained within trimester-specific reference ranges, subclinical hypothyroidism was identified in 16.3% of participants, indicating thyroidal stress in a subset of the cohort (Table 2). This coexistence of low iodine status and the absence of frank thyroid failure reflects the thyroid gland’s potent adaptive capacity—such as increased iodine uptake efficiency and preferential triiodothyronine secretion—which operates to maintain euthyroidism even under conditions of nutritional strain [2,6]. However, the notable prevalence of subclinical hypothyroidism suggests that this adaptive reserve is compromised in a significant proportion of pregnant women. Consistent with this complex physiology, urinary iodine indicators (UIC, UIC/UCr, and 24h-UIE) did not differ significantly between women with and without subclinical hypothyroidism in the overall cohort. However, a trimester-specific analysis revealed a crucial distinction: specifically in the first trimester—a period of maximal fetal dependence on maternal thyroxine—the median 24h-UIE was actually significantly higher in women with subclinical hypothyroidism compared to euthyroid women (*p* = 0.037). While seemingly paradoxical, an examination of the absolute values perfectly illustrates the fundamental physiological lag between acute intake markers and chronic functional markers. Although the median in the SCH group (134.2 µg/day) remained marginally below the EAR adequacy threshold, the data revealed a wide distribution. Several individuals in this group exhibited robust, adequate 24h-UIE levels, likely reflecting sudden dietary compensations (e.g., abruptly increased consumption of dairy or iodized salt) immediately upon pregnancy confirmation. However, because maternal thyroid adaptation requires a physiological lag time of several weeks, their TSH levels still reflect the pre-existing state of chronic iodine deficiency (manifesting as SCH) and have not yet normalized. This highlights precisely why a single acute spike in urinary excretion cannot instantaneously resolve chronic thyroid stress. The fact that 24h-UIE captured this acute variance—while spot indices did not—highlights its heightened sensitivity to sudden dietary shifts in early pregnancy, rather than indicating absolute diagnostic superiority for chronic thyroid status. This overarching lack of strong correlation supports the view that short-term urinary indices primarily reflect recent intake and may not accurately capture the chronic deprivation—or the subsequent adaptation lag time—required to alter thyroid function [9,10]. Moreover, population-level iodine insufficiency can clearly coexist with largely compensated thyroid function, underscoring the clinically silent nature of this vulnerability and the critical necessity for prevention-oriented monitoring [30].

Our analyses suggest that UIC/UCr significantly outperforms unadjusted spot UIC in approximating the 24h-UIE reference standard in this pregnancy sample. Notably, this advantage was most evident in the second trimester, where the correlation between spot UIC and 24h-UIE was entirely lost (rho = 0.125, *p* = 0.275). This mid-pregnancy loss of correlation is highly consistent with the significant peak in estimated glomerular filtration rate (eGFR) observed during this period, which exacerbates urine dilution and renders unadjusted spot UIC unreliable. In stark contrast, UIC/UCr successfully corrected for these fluctuations, maintaining a strong and significant association with 24h-UIE (rho = 0.553, *p* < 0.001). Beyond these correlations, our strictly EAR-adjusted ROC analysis provided definitive evidence of this diagnostic superiority: UIC/UCr yielded a significantly greater area under the curve compared to spot UIC alone (0.774 vs. 0.670; DeLong test, *p* = 0.016) in identifying adequate individual iodine intake. These compelling results are highly consistent with the findings of Li et al. [13], who similarly demonstrated a clear diagnostic advantage for UIC/UCr over spot UIC (AUC 0.92 vs. 0.61). Taken together, these robust physiological and diagnostic findings substantiate concerns regarding the confounding effects of pregnancy-induced urine dilution [8,11,12,13,31], demonstrating that creatinine adjustment is essential to improve the interpretability and accuracy of spot iodine measures when 24 h collection is unfeasible.

While our findings highlight the diagnostic advantages of the creatinine-adjusted index, it is important to acknowledge that creatinine correction itself has inherent limitations. In pregnancy, where creatinine generation and renal handling undergo significant shifts across gestation, UIC/UCr should be interpreted cautiously and within the broader clinical context rather than as a universally flawless metric [8,11]. Nevertheless, as reflected in our data and supported by recent longitudinal studies, UIC/UCr offers notably greater stability than unadjusted spot UIC by correcting for the progressive volume expansion and increased glomerular filtration that characterize advancing gestation [31,32]. Taken together, our results position UIC/UCr as a robust and necessary complementary marker to spot UIC when 24 h collections are unfeasible, while simultaneously recognizing the ongoing scholarly debate regarding the optimal metric for iodine assessment in pregnancy [9,12,13].

Our finding that 96.5% of pregnant women regularly consume dairy products is a noteworthy observation. As highlighted in the literature, dairy products can serve as a major source of dietary iodine, sometimes even surpassing the contribution of iodized salt in populations with high dairy intake. This substantial dietary contribution is largely attributed to the routine supplementation of livestock with iodine-containing feeds and the use of iodophor sanitizers during milking—standardized modern agricultural practices that significantly enhance the iodine content of dairy products [33,34]. Finally, the observed knowledge gaps regarding iodine sources and salt practices align with global reports of suboptimal maternal awareness [14,16,35]. Given that dietary behaviors significantly influence iodine exposure even in mandatory iodization settings [15,16], integrating structured iodine education into routine antenatal care represents a pragmatic, scalable intervention to complement ongoing biochemical surveillance.

Strengths of this study include the dual urine-collection protocol, allowing direct comparison of spot indices with measured 24h-UIE, alongside a combined biochemical and behavioral assessment to contextualize iodine status. However, certain limitations must be acknowledged. The cross-sectional design precludes causal inference, and the regional focus on a single province may limit the generalizability of the findings to the broader population. Furthermore, while 24 h urine collection is superior to spot sampling, a single collection reflects only recent intake; thus, inherent intra-individual variability remains a challenge when estimating habitual iodine status [9,10,11]. Additionally, the relatively limited number of iodine-adequate participants likely constrained the statistical power for ROC-based discrimination and comparisons of AUC values, particularly when contrasted with larger multi-center cohorts [13]. Specifically, although a significant association was identified in the first trimester, the small sample size of the subclinical hypothyroidism subgroup in this period necessitates caution in interpreting the findings. Finally, the lack of direct quantitative measurement of iodine concentration in the household salt remains a limitation, as actual iodine content may vary based on storage and handling practices.

## 5. Conclusions

In conclusion, this study reveals a high prevalence of iodine inadequacy among pregnant women in Afyonkarahisar, Turkey, robustly confirmed at both the population level via WHO median thresholds and using the EAR-adjusted 24h-UIE criteria. Crucially, our findings demonstrate that the creatinine-adjusted UIC/UCr index exhibits superior diagnostic accuracy compared to spot UIC alone in identifying adequate recent iodine intake. This establishes UIC/UCr as a highly reliable and practical alternative marker when 24 h urine collection is unfeasible, effectively adjusting for variable urine dilution. Furthermore, given the substantial gaps identified in maternal knowledge, these results underscore an urgent clinical need for targeted antenatal iodine education and the implementation of continuous, methodologically precise surveillance of iodine nutrition during pregnancy.

## Figures and Tables

**Table 1:** Consumption frequency and maternal knowledge regarding iodine content of selected food items (*n* = 227).

Food Item	Consumed at Least Weekly *n* (%)	Knowledge: “I Don’t Know” *n* (%)
Goitrogenic Foods		
Turnip	8 (3.5)	207 (91.2)
Radish	76 (33.5)	202 (89.0)
Cabbage	32 (14.1)	197 (86.8)
Black cabbage	26 (11.5)	197 (86.8)
Broccoli	60 (26.4)	194 (85.5)
Cauliflower	68 (30.0)	196 (86.3)
Millet	65 (28.6)	194 (85.5)
Dietary Iodine Sources		
Dairy products	219 (96.5)	186 (81.9)
Green vegetables	219 (96.5)	181 (79.7)
Meat products	191 (84.1)	184 (81.1)
Legumes	183 (80.6)	181 (79.7)
Seafood	107 (47.1)	169 (74.4)

“Consumed at least weekly” indicates consumption “everyday” or “a few days a week”. Daily seafood consumption was 0%.

**Table 2:** Comparison of thyroid function, renal parameters, and urinary iodine indices stratified by pregnancy trimester.

Parameter	1st Trimester (*n* = 63)	2nd Trimester (*n* = 78)	3rd Trimester (*n* = 86)	Overall (*n* = 227)	*p*
Renal and thyroid parameters					
UCr (mg/dL)	95.2 (65.6–140.8) ^a^	95.4 (50.5–145.5) ^ab^	68.3 (41.8–115) ^b^	87.5 (51.3–136.6)	**0.008**
eGFR (mL/min/1.73 m^2^)	141.5 (125.4–156.3) ^a^	157.6 (143.7–179.7) ^b^	160.3 (142.9–176.1) ^b^	154.3 (138.4–175.7)	**<0.001**
TSH (mU/L)	1.80 (1.1–2.5) ^a^	2.05 (1.4–2.6) ^ab^	2.24 (1.6–2.9) ^b^	2.05 (1.3–2.7)	**0.012**
fT4 (ng/dL)	1.23 (1.1–1.3) ^a^	1.05 (0.9–1.1) ^b^	1.01 (0.9–1.1) ^b^	1.07 (0.9–1.2)	**<0.001**
Subclinical hypothyroidism *n* (%)	14 (22.2)	7 (9.0)	16 (18.6)	37 (16.3)	0.081 ^†^
Iodine indices					
UIC (µg/L)	67.5 (39–102)	71.6 (37.9–95.1)	53.2 (36.3–80.7)	59.0 (38.6–93)	0.121
UIC/UCr (µg/g)	66.8 (40.8–104.6)	71.5 (46.8–111.1)	79.7 (54.8–121.1)	72.7 (45.9–111.1)	0.314
24h-UIE (µg/day)	107.3 (75.1–149.1)	118.8 (88.5–171.9)	108.0 (72.0–157.1)	111.2 (76.6–162.3)	0.302

Data are presented as median (25th–75th percentiles) unless otherwise indicated. Statistically significant *p*-values (*p* < 0.05) are highlighted in bold. *p* values for continuous variables are from Kruskal–Wallis tests. ^†^ *p* value for subclinical hypothyroidism prevalence is from chi-square test comparing proportions across trimesters (*df* = 2). Different superscript letters indicate statistically significant differences in Bonferroni-corrected pairwise comparisons (post hoc). Trimester groups were independent (cross-sectional). Abbreviations: UIC: spot urinary iodine concentration; UCr: spot urinary creatinine; UIC/UCr: urinary iodine-to-creatinine ratio; 24h-UIE: 24 h urinary iodine excretion; eGFR: estimated glomerular filtration rate; TSH: thyroid-stimulating hormone; fT4: free thyroxine.

**Table 3:** Comparison of median urinary iodine parameters between euthyroid and subclinical hypothyroidism groups stratified by trimester.

Trimester & Thyroid Status	*n* (%)	Spot UIC (µg/L) Median (Q1–Q3)	UIC/UCr (µg/g) Median (Q1–Q3)	24h-UIE (µg/Day) Median (Q1–Q3)
1st Trimester				
Euthyroid	49 (77.8%)	69.0	68.7	100.3
		(38.0–103.6)	(39.6–95.2)	(67.2–140.2)
Subclinical	14 (22.2%)	54.6	60.5	134.2
Hypothyroidism		(38.6–105.1)	(44.7–108.7)	(97.1–194.1)
*p*-value		0.856	0.574	**0.037**
2nd Trimester				
Euthyroid	71 (91.0%)	69.6	71.9	117.7
		(35.6–91.5)	(47.4–111.1)	(88.2–171.8)
Subclinical	7	102.5	53.0	132.8
Hypothyroidism	(9.0%)	(67.6–104.1)	(44.8–216.4)	(102.5–225.7)
*p*-value		0.181	0.993	0.582
3rd Trimester				
Euthyroid	70 (81.4%)	51.7	75.2	108.0
		(36.3–70.8)	(57.1–103.8)	(71.7–156.7)
Subclinical	16 (18.6%)	69.4	109.9	110.8
Hypothyroidism		(33.7–132.8)	(43.1–137.9)	(81.4–207.7)
*p*-value		0.126	0.297	0.523
Overall				
Euthyroid	190 (83.7%)	57.4	72.7	109.6
		(37.0–90.1)	(46.6–102.3)	(74.3–156.7)
Subclinical	37 (16.3%)	71.7	79.6	116.7
Hypothyroidism		(39.3–108.3)	(45.4–123.3)	(93.1–194.7)
*p*-value		0.198	0.423	0.091

Abbreviations: UIC: Urinary Iodine Concentration; 24h-UIE: 24-h Urinary Iodine Excretion; UIC/UCr: Urinary Iodine-to-Creatinine Ratio; Q1: First quartile (25th percentile); Q3: Third quartile (75th percentile). Comparisons were performed using the Mann–Whitney U test. Statistically significant ***p***-values (***p*** < 0.05) are highlighted in bold.

**Table 4:** Spearman correlations of urinary iodine indices with 24 h urinary iodine excretion (24h-UIE) and renal function (*n* = 227).

Variable Pair	Overall (*n* = 227)	1st Trimester (*n* = 63)	2nd Trimester (*n* = 78)	3rd Trimester (*n* = 86)
Correlation with 24h-UIE (reference for recent iodine intake)				
UIC vs. 24h-UIE	**0.270 (<0.001)**	**0.302 (0.016)**	0.125 (0.275)	**0.343 (0.001)**
UIC/UCr vs. 24h-UIE	**0.491 (<0.001)**	**0.394 (0.001)**	**0.553 (<0.001)**	**0.533 (<0.001)**
Renal and dilution-related associations				
UIC vs. UCr	**0.489 (<0.001)**	**0.367 (0.003)**	**0.451 (<0.001)**	**0.578 (<0.001)**
UIC/UCr vs. UCr	**−0.521 (<0.001)**	**−0.544 (<0.001)**	**−0.525 (<0.001)**	**−0.463 (<0.001)**
UCr vs. 24h-UIE	**−0.202 (0.002)**	−0.091 (0.479)	**−0.416 (<0.001)**	−0.100 (0.360)
eGFR vs. UCr	**−0.192 (0.004)**	0.008 (0.948)	**−0.365 (0.001)**	−0.067 (0.542)
BMI vs. 24h-UIE	0.050 (0.452)	0.110 (0.392)	−0.104 (0.363)	0.167 (0.124)

Values are Spearman’s rho (*p*-value). Statistically significant *p*-values (*p* < 0.05) are highlighted in bold. Abbreviations: UIC: spot urinary iodine concentration; UCr: spot urinary creatinine concentration; UIC/UCr: urinary iodine-to-creatinine ratio; 24h-UIE: 24 h urinary iodine excretion; eGFR: estimated glomerular filtration rate; BMI: body mass index.

## Data Availability

The data presented in this study are available on request from the corresponding author. The data are not publicly available due to privacy and ethical restrictions.

## Abbreviations

The following abbreviations are used in this manuscript:

24h-UIE	24 h Urinary Iodine Excretion
ANOVA	Analysis of Variance
AUC	Area Under the Curve
BMI	Body Mass Index
CI	Confidence Interval
ECLIA	Electrochemiluminescence Immunoassay
eGFR	Estimated Glomerular Filtration Rate
FHC	Family Health Center
fT4	Free Thyroxine
ICP–MS	Inductively Coupled Plasma–Mass Spectrometry
IQR	Interquartile Range
MDRD	Modification of Diet in Renal Disease
ROC	Receiver Operating Characteristic
SD	Standard Deviation
STROBE	Strengthening the Reporting of Observational Studies in Epidemiology
T4	Thyroxine
TSH	Thyroid-Stimulating Hormone
UCr	Urinary Creatinine
UIC	Urinary Iodine Concentration
UIC/UCr	Urinary Iodine-to-Creatinine Ratio
UNICEF	United Nations Children’s Fund
WHO	World Health Organization
